# Risk factors for mechanical complications in degenerative lumbar scoliosis with concomitant thoracolumbar kyphosis: does the selection of the upper instrumented vertebra matter?

**DOI:** 10.1186/s13018-025-05458-z

**Published:** 2025-01-23

**Authors:** Weipeng Qiu, Zhuoran Sun, Ze Chen, Siyu Zhou, Qiang Qi, Weishi Li

**Affiliations:** 1 Department of Orthopaedics, Peking University Third Hospital, 49 North Garden Road, Haidian District, Beijing, 100191 China; 2 Beijing Key Laboratory of Spinal Disease Research, Beijing, China; 3 Engineering Research Center of Bone and Joint Precision Medicine, Ministry of Education, Beijing, China

**Keywords:** Thoracolumbar kyphosis, Degenerative lumbar scoliosis, Mechanical complication, Upper instrumented vertebrae, Risk factor

## Abstract

**Background:**

Degenerative lumbar scoliosis (DLS) represents a distinct subset of adult spinal deformity, frequently co-occurring with thoracolumbar kyphosis (TLK) in the sagittal plane. TLK is typically viewed as detrimental in degenerative spinal conditions and has been linked to increased pain severity and a higher prevalence of mechanical complications (MC) as previously reported. The present study aimed to identify the risk factors associated with the development of MC in patients with DLS and concomitant TLK.

**Methods:**

This study retrospectively included 65 DLS patients with a TLK > 20°. During follow-up, MC events were recorded, and patients were categorized into MC and non-MC groups. Spinopelvic parameters were assessed pre- and post-surgery. The locations of the kyphotic apex (KA) and the upper instrumented vertebra (UIV) were documented. Clinical and radiographic data were compared between the two groups. A multivariate logistic regression model was employed to analyze the independent risk factors for MC.

**Results:**

Twenty-nine (44.6%) patients developed at least one MC at final follow-up. At baseline, patients in the MC group exhibited lower bone mineral density (*P* = 0.002), lower distal lumbar lordosis (DLL, *P* = 0.025), and higher sagittal vertical axis (SVA, *P* = 0.005). Post-surgery, the KA shifted cranially with an average vertebral displacement of 2.6 ± 1.8. The proportion of UIV located in the postoperative KA area significantly increased (*P* < 0.001). The MC group had a higher incidence of Roussouly type mismatch (*P* = 0.023) and UIV located in the KA area (*P* = 0.003). Multivariate logistic regression analysis revealed that UIV located in the KA area (OR = 3.798, *P* = 0.043), increased preoperative SVA (OR = 1.017, *P* = 0.016) and osteoporosis (OR = 6.713, *P* = 0.007) were independent risk factors for MC.

**Conclusions:**

The presence of UIV in the KA area, preoperative sagittal imbalance, and osteoporosis were identified as significant risk factors for MC in patients with DLS and concomitant TLK. The spinal morphological characteristics of TLK must be taken into account during surgical planning to prevent placing the UIV within the kyphotic region.

## Background

Mechanical complications (MC) are frequently associated with suboptimal clinical outcomes and serve as a primary driver of revision surgery in the field of adult spinal deformity (ASD) [1]. Multiple risk factors of MC have been identified in the literature, including older age, obesity, osteoporosis, preoperative sagittal imbalance, overcorrection of lumbar lordosis (LL), extending fusion to the pelvic and upper instrumented vertebra (UIV) located at the thoracolumbar junction [2–7]. However, ASD is a highly heterogeneous spinal disorder, and the characteristics of its subtypes should not be neglected to ensure the precise formulation of surgical strategies [8–10].

For patients with degenerative lumbar scoliosis (DLS), the most prevalent form of ASD, increasing axial rotation produced by scoliosis may induce a rotational kyphosis in the thoracolumbar junction. Therefore, the coexistence of thoracolumbar kyphosis (TLK) in DLS patients is not uncommon in clinical practice. Typically, TLK is considered as detrimental in degenerative spinal conditions. Previous studies have showed that the presence of TLK was correlated with the severity of pain and contributed to a higher incidence of MC in degenerative scoliosis [11–14]. In particular, the selection of UIV emerges as a challenging issue in the context of TLK. It has been recommended to extend proximal fusion to the upper thoracic spine or at least above T10 if TLK exceeds 20° [15–18]. However, the aforementioned criteria for UIV selection are subjective and do not take into account the morphological characteristics of TLK.

In patients with DLS and concomitant TLK, the formation of regional kyphosis could increase the stress in the thoracolumbar junction area [14], a biomechanical transition zone from the less mobile thoracic spine to the lumbar spine. In the sagittal plane, the kyphotic apex (KA) is almost located in this area. Consequently, the selection of the UIV inevitably requires consideration of its positional relationship with the KA, if long-segment fusion to the upper thoracic spine is not opted for. Therefore, we hypothesized that the selection of UIV might have a more substantial impact on the development of MC for this specific subgroup of patients.

The objective of this study was to identify potential risk factors linked to the development of MC, and to clarify the influence of UIV selection in patients with DLS and concomitant TLK.

## Methods

### Patient population

This study is a retrospective case series reviewing 361 consecutive patients with DLS who underwent surgical treatment at our institution from January 2012 to December 2022. Cases with concomitant TLK were selected in this study. Inclusion criteria were: (1) Cobb angle > 20°; (2) TLK (T10-L2) > 20°; (3) age at surgery > 45 years; (4) posterior long-segment instrumentation and fusion surgery (≥ 4 vertebrae); (5) with UIV located at the lower thoracic spine or thoracolumbar junction; (6) at least a 2-year follow-up; (7) complete clinical and imaging data before and after surgery. Exclusion criteria were: (1) history of previous spinal surgery; (2) other types of scoliosis such as adult idiopathic scoliosis, congenital scoliosis, and neuromuscular scoliosis; and (3) concomitant spinal tumors, spinal tuberculosis, spinal trauma and ankylosing spondylitis. All patients underwent a full pedicle screw internal fixation system without the use of transverse hooks and laminar hooks at the UIV.

### Surgical procedure

Under general anesthesia, all patients were placed prone on a radiolucent operating table. A posterior midline incision was made for conventional exposure. Pedicle screws were inserted according to the surgical planning. Multilevel posterior column osteotomy (PCO) plus unilateral cage strutting was performed for the correction of kyphoscoliosis. The posterior elements, encompassing the spinous process, bilateral lamina, and the adjacent facet joints, were removed. The decompressive release was performed bilaterally. Using a sequential reamer, kyphoscoliosis and disc height were corrected. The smallest reamer was horizontally inserted into the narrowed disc space on the concave side and rotated 90°, restoring disc height and reducing scoliosis. The maneuver was repeated using a larger reamer, further enlarging the disc space. A rod with slightly hypo-lordotic contouring was put on the concave side and distracted. A lordotic-shaped cage filling with autologous bone graft was introduced into concave side of the disc space. The second rod with slightly hyper-lordotic contouring was put on the convex side and further compressed. For rigid deformity, asymmetrical pedicle subtraction osteotomy (PSO) was performed to correct the kyphoscoliosis.

### Data collection

Demographic data was recorded including age, gender, height, weight, body mass index (BMI), bone mineral density (BMD, T-score less than -2.5 was diagnosed as osteoporosis) and follow-up duration. Surgical data included fused level, location of UIV and lowest instrumented vertebrae (LIV), osteotomy method, operative time and blood loss. MC were recorded during the follow-up using similar criteria to those reported by Yilgor et al. [2], including proximal junctional kyphosis (PJK), proximal junctional failure (PJF), distal junctional kyphosis or failure (DJK/F), rod fracture and screw breakage/ pull-out. PJK was defined as the proximal junctional angle (PJA) ≥ 10° during follow-up and at least 10° greater than the preoperative measurement [19]. PJF was defined as a vertebral fracture located at the UIV/UIV + 1 level, a failure of proximal fixation, or a need for proximal revision surgery. The patient-reported outcomes were evaluated before surgery and at the final follow-up using the following clinical scoring systems: the visual analogue scale (VAS) for back pain and leg pain, the Oswestry disability index (ODI), and the Japanese Orthopedic Association (JOA) score.

### Radiographic measurements

All patients were required to take standard standing anteroposterior and lateral full spine X-rays preoperatively, 3 month postoperatively, and at the final follow-up. The radiographic data were measured by two experienced spinal surgeons using the Picture Archiving Communications System (Centricity RIS/PACS, GE healthcare).

Spinopelvic parameters were measured including Cobb angle of the main curve, sacral slope (SS), pelvic tilt (PT), pelvic incidence (PI), lumbar lordosis (LL, L1-S1), distal lumbar lordosis (DLL, L4-S1), TLK (T10-L2), thoracic kyphosis (TK, T4-T12), sagittal vertical axis (SVA), T1 pelvic angle (TPA) and PJA (the angle between the superior endplate of UIV + 2 and the inferior endplate of UIV). Supine TLK was also measured in lateral computed tomography (CT) scout preoperatively to assess flexibility of the thoracolumbar junction compared with standing TLK. TLK flexibility was calculated using the following formula: (Standing TLK—Supine TLK) / Standing TLK × 100%.

The KA was identified by the plumb line in the sagittal plane. The definition of KA migration referred to the number of vertebrae by which the postoperative KA shifted cranially relative to the preoperative location. The KA area was defined as the segment within one vertebra adjacent to the KA. If the UIV level is within KA ± 1 vertebra, the UIV located in the KA area is considered. Sagittal profiles were also documented, including lordotic apex (LA), inflection point, number of vertebrae in the lordosis and the spinal shape. The current Roussouly type was identified based on the postoperative sagittal profiles. Roussouly type mismatch was determined if these profiles did not match the ideal sagittal profiles corresponding to PI value, as reported previously in the literature [20–22].

### Statistical analysis

SPSS 26.0 (IBM, USA) statistical software was used to analyze the data. Continuous variables were expressed as mean ± standard deviation, and categorical variables were expressed as frequency (percentage). Independent samples t-test or Mann–Whitney U test were used for between-group comparison of continuous variables, while χ2 test or Fisher test were used for between-group comparison of categorical variables. Logistic regression analysis was employed to determine the risk factors for MC. Statistical significance was set at a level of *P* < 0.05.

## Results

### Baseline data

**Fig. 1:**
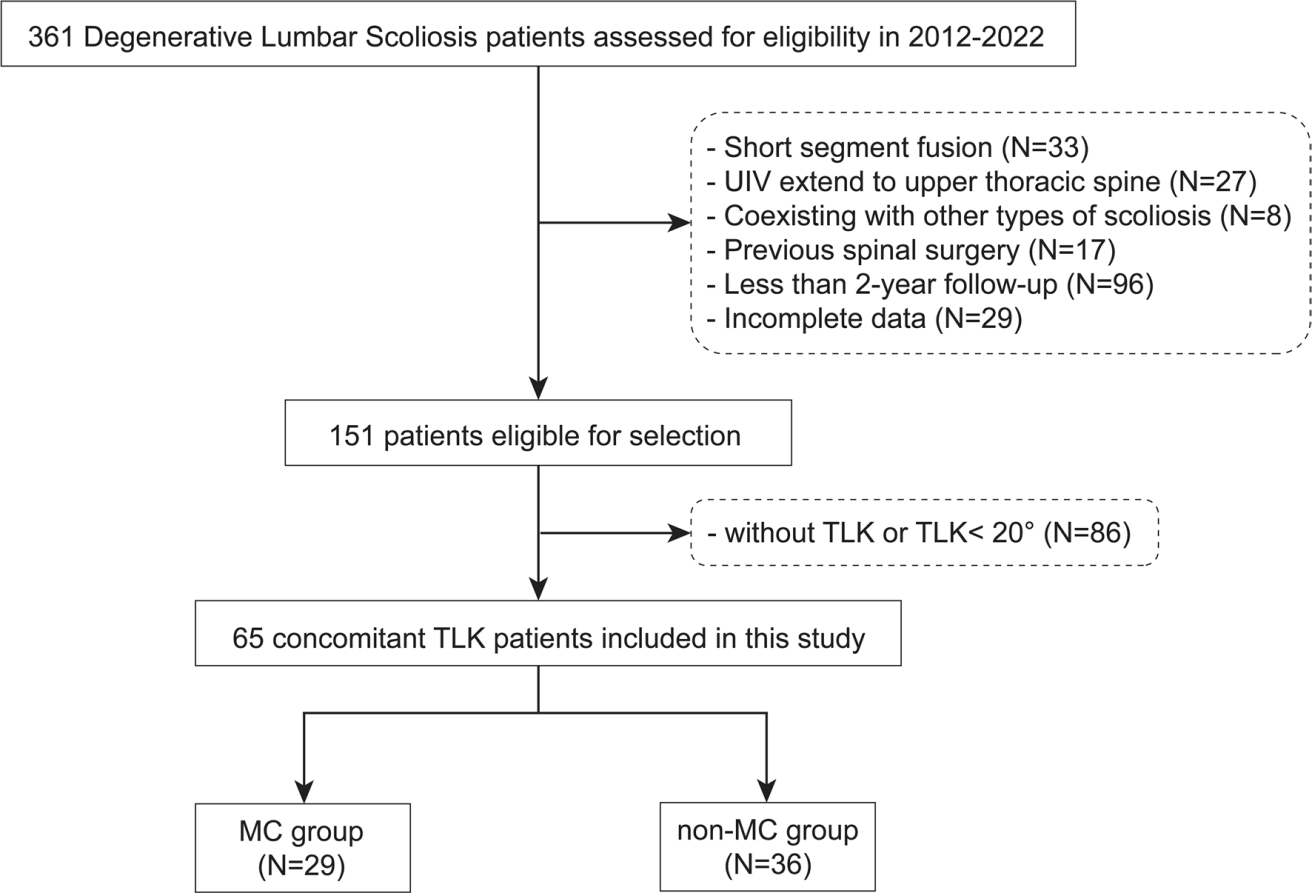
Flow diagram of patient enrollment

A total of 65 DLS patients with TLK were included in the study, including 13 males and 52 females (Fig. 1). The mean age was 62.9 ± 7.1 years (range 50–80 years). The mean BMI was 26.2 ± 3.6 kg/m^2^. The average follow-up duration was 39.1 ± 20.1 months. The mean fused level was 6.5 ± 2.1. During follow-up, 29 (44.6%) patients suffered at least one MC (27 PJK/PJF, 2 DJF, 2 rod fracture, 3 screw breakage/ pull-out), with worse patient-reported outcomes. In the MC group, 4 cases underwent revision surgery. Table 1 summarized the demographic and surgical data of the MC and Non-MC groups.

**Table 1:** Demographic and clinical data of patients with or without mechanical complications

Variables	MC group (*n* = 29)	Non-MC group (*n* = 36)	*P*-value
Age (yr)	64.2 ± 6.2	61.8 ± 7.7	0.190
Gender (M/F)	3/26	10/26	0.081
BMI (kg/m2)	25.7 ± 3.4	26.6 ± 3.7	0.325
BMD (T-score)	– 2.1 ± 1.6	– 0.3 ± 2.2	0.002*
Follow-up duration (mo)	42.7 ± 20.2	36.2 ± 19.9	0.200
Fused level	6.9 ± 2.3	6.2 ± 1.9	0.187
Operative time (min)	289.3 ± 81.6	289.6 ± 67.2	0.986
Blood loss (mL)	1343.8 ± 1450.1	1291.7 ± 885.3	0.859
Hospital days (D)	12.8 ± 4.7	12.3 ± 4.6	0.692
ASA (I: II: III)	1:26:2	4:29:3	0.490
Osteotomy (PCO/PSO)	24/5	30/6	0.951
Preop VAS (back)	5.8 ± 2.2	5.7 ± 2.2	0.786
Preop VAS (leg)	4 ± 2.9	4.5 ± 2.9	0.483
Preop ODI	52 ± 17.2	54.1 ± 15.6	0.622
Preop JOA	14.8 ± 5.3	13.8 ± 5.2	0.467
Follow-up VAS (back)	4.3 ± 2.4	2.3 ± 1.6	< 0.001*
Follow-up VAS (leg)	3.3 ± 2.3	1.8 ± 1.7	0.006*
Follow-up ODI	38.9 ± 23.7	20.7 ± 15.8	0.001*
Follow-up JOA	17.7 ± 5.8	23.1 ± 3.9	< 0.001*

BMI indicates body mass index; *BMD* bone mineral density, *ASA* American Society of Anesthesiologists, *PCO* posterior column osteotomy, *PSO* pedicle subtraction osteotomy, *VAS* Visual Analogue Scale, *ODI* Oswestry Disability Index, *JOA* Japanese Orthopedic Association

^*^Means statistical significance

### Comparison between MC and Non-MC groups

At baseline, the patients in the MC group had lower BMD compared to the non-MC group (*P* = 0.002). The comparison of preoperative radiographic parameters showed that the patients in the MC group had lower DLL (*P* = 0.025) and higher SVA (*P* = 0.005) than the non-MC group **(**Table 2**)**. Comparisons of radiographic parameters after surgery between the two groups are shown in Table 3. No statistically significant differences were observed in any parameter 3 month postoperatively. At the final follow-up, the patients in the MC group had greater PT (*P* = 0.037), SVA (*P* = 0.001), TPA (*P* = 0.007) and PJA (*P* < 0.001) than the non-MC group.

**Table 2:** Comparisons of radiographic parameters between the patients with and without mechanical complications before surgery

Variables	MC group (*n* = 29)	Non-MC group (*n* = 36)	*P* value
Cobb (°)	34.2 ± 10.0	30.7 ± 13.0	0.235
SS (°)	18.6 ± 10.0	23.8 ± 10.9	0.055
PT (°)	29.0 ± 9.9	24.9 ± 10.5	0.115
PI (°)	47.6 ± 10.7	48.6 ± 12.8	0.733
LL (°)	21.6 ± 19.6	27.9 ± 21.4	0.224
DLL (°)	29.0 ± 13.0	35.8 ± 11.0	0.025*
TLK (°)	29.5 ± 12.8	33.7 ± 12.0	0.173
TLK flexibility (%)	48.6 ± 21.7	42.9 ± 25.0	0.334
TK (°)	24.4 ± 18.2	24.0 ± 19.3	0.925
PI-LL (°)	26.0 ± 18.8	20.7 ± 18.2	0.255
SVA (mm)	76.2 ± 47.6	44.0 ± 42.2	0.005*
TPA (°)	24.5 ± 13.6	20.8 ± 10.3	0.236
PJA (°)	6.7 ± 4.2	8.6 ± 9.8	0.278

^*^Means statistical significance

**Table 3:** Comparisons of radiographic parameters between the patients with and without mechanical complications after surgery

Variables	Postoperative		Final follow-up			
	MC group	Non-MC group	*P *value	MC group	Non-MC group	*P* value
Cobb (°)	11.9 ± 5.7	9.8 ± 5.9	0.158	14.0 ± 7.0	11.1 ± 5.5	0.064
SS (°)	25.7 ± 7.5	27.9 ± 9.1	0.284	20.8 ± 8.3	24.8 ± 9.6	0.08
PT (°)	22.5 ± 9.1	19.7 ± 9.9	0.250	28.5 ± 10.3	23.3 ± 9.1	0.037*
PI (°)	48.1 ± 10.2	47.6 ± 12.1	0.863	48.9 ± 10.2	48.3 ± 11.9	0.826
LL (°)	35.2 ± 10	38.5 ± 13.1	0.274	30.3 ± 14.2	34.9 ± 15.1	0.214
DLL (°)	25.5 ± 8.6	29.5 ± 8.2	0.061	24.5 ± 11.6	29.4 ± 11.7	0.100
TLK (°)	14.8 ± 7.6	16.1 ± 11.1	0.587	20.3 ± 11.6	19.1 ± 11.2	0.677
TK (°)	27.3 ± 9.5	27.2 ± 13.9	0.973	36.2 ± 15.3	30.0 ± 15.1	0.105
PI-LL (°)	13.0 ± 11.9	9.1 ± 9.9	0.160	18.6 ± 15.8	13.4 ± 10.8	0.136
SVA (mm)	34.6 ± 40.9	17.6 ± 34.2	0.072	76.5 ± 53.9	36.6 ± 35.5	0.001*
TPA (°)	17.7 ± 10.4	13.9 ± 8.8	0.118	24.7 ± 11.4	17.9 ± 8.1	0.007*
PJA (°)	12.9 ± 5.7	10.3 ± 9.1	0.164	21.3 ± 6.1	12.2 ± 9.1	< 0.001*

^*^Means statistical significance

Table 4 showed the variations of sagittal morphology before and after surgery. Totally, preoperative KA were mainly located at T11 or T12 (*n* = 36), followed by L1 or L2 (*n* = 27) and T10 (*n* = 2). After surgery, the mean KA migration was 2.6 ± 1.8 (range 0–7). Postoperative KA were mainly located at T10 or above (*n* = 38), followed by T11 or T12 (*n* = 25) and L1 or L2 (*n* = 2). Following the KA migration, the proportion of UIV located in the KA area increased significantly from 29.2% (relative to preoperative KA) to 63.1% (relative to postoperative KA) (*P* < 0.001). Regarding the intergroup comparisons, no significant differences were found in KA distribution, LA distribution, KA migration and LIV location between the two groups. However, the incidence of Roussouly type mismatch (*P* = 0.023) and UIV located in the postoperative KA area (*P* = 0.003) were significantly higher in the MC group.

**Table 4:** Variations of sagittal apex and locations of instrumentation vertebrae

Variables	MC group (*n* = 29)	Non-MC group (*n* = 36)	*P *value
Preop KA distribution			0.183
T10 or above	0 (0)	3 (8.3%)	
T11 or T12	14 (48.3%)	22 (61.1%)	
L1 or L2	15 (51.7%)	12 (33.3%)	
Postop KA distribution			0.612
T10 or above	15 (51.7%)	23 (63.9%)	
T11 or T12	13 (44.8%)	12 (33.3%)	
L1 or L2	1 (3.4%)	1 (2.8%)	
KA migration (vertebrae)	2.7 ± 2.0	2.58 ± 1.8	0.622
Postop LA distribution			0.363
L5	7 (24.1%)	12 (33.3%)	
L4-L5	13 (44.8%)	10 (27.8%)	
L4	6 (20.7%)	12 (33.3%)	
L3-4	3 (10.3%)	2 (5.6%)	
Roussouly type mismatch	17 (58.6%)	11 (30.6%)	0.023*
UIV location			
Located in the preop KA area	8 (27.6%)	11 (30.6%)	0.794
Located in the postop KA area	24 (82.8%)	17 (47.2%)	0.003*
LIV location			0.488
L5 or above	12 (41.4%)	18 (50.0%)	
S1 or pelvic	17 (58.6%)	18 (50.0%)	

KA indicates kyphotic apex; *LA* lordotic apex, *UIV* upper instrumentation vertebrae, *LIV* lowest instrumentation vertebrae

^*^Means statistical significance

### Analysis of risk factors for mechanical complications

In the univariate analysis, osteoporosis (*P* = 0.0016), UIV located in the KA area (*P* = 0.005), Roussouly type mismatch (*P* = 0.025), lower preoperative DLL (*P* = 0.031) and greater preoperative SVA (*P* = 0.009) were identified as risk factors for MC **(**Table 5**)**. Candidate risk factors with* P* < 0.01 in the univariate analysis were included in multivariate logistic regression analysis with backward elimination. After controlling for confounders, the model showed that the main variables that independently impacted MC occurrence included UIV located in the KA area (OR = 3.798, *P* = 0.043), increased preoperative SVA (OR = 1.017, *P* = 0.016) and osteoporosis (OR = 6.713, *P* = 0.007).

**Table 5:** Independent risk factor of mechanical complications identified by logistic regression

Variables	Univariate analysis			Multivariate analysis		
	OR	95% CI	*P*-value	OR	95% CI	*P*-value
Age	1.049	0.977–1.126	0.189	Not selected		
Gender	3.333	0.822–13.518	0.092	Not selected		
BMI	0.931	0.808–1.072	0.321	Not selected		
Osteoporosis (yes/no)	4.376	1.319–14.520	0.016*	6.713	1.667–27.031	0.007*
Fused level	1.177	0.924–1.498	0.187	Not selected		
UIV located in the KA area (yes/no)	5.365	1.674–17.194	0.005*	3.798	1.040–13.874	0.043*
Roussouly type mismatch (yes/no)	3.220	1.156–8.966	0.025*	Not selected		
Postop PI-LL	1.035	0.986–1.085	0.164	Not selected		
Postop SVA	1.013	0.998–1.028	0.082	Not selected		
Preop DLL	0.952	0.910–0.996	0.031*	Not selected		
Preop SVA	1.016	1.004–1.029	0.009*	1.017	1.003–1.031	0.016*

BMI indicates body mass index; *UIV* upper instrumentation vertebrae, *KA* kyphosis apex, *DLL *distal lumbar lordosis, *SVA* sagittal vertical axis

^*^Means statistical significance

## Discussion

As a regional sagittal modifier, TLK was initially proposed in the SRS classification for ASD [23], while disappeared in the SRS-Schwab classification [24]. In the Roussouly classification, TLK was associated with type 1 morphology, characterized by a short lordotic curve with a low inflection point [21, 25]. However, thus far, the existing classification systems provide insufficient guidance on how to address TLK in surgical planning.

The current study focused on the surgical outcomes of DLS patients with concomitant TLK. The prevalence of MC is shown to be high (44.6%) in this cohort of patients. The occurrence of this adverse event has led to a significant decline in clinical function improvement and quality of life among patients in the MC group. Our results showed that UIV located in the KA area, Roussouly type mismatch, lower preoperative DLL, higher preoperative SVA and osteoporosis significantly increased the incidence of MC for patients with DLS and concomitant TLK. However, after controlling for confounders, multivariate analysis showed that the three main risk factors were UIV located in the KA area, higher preoperative SVA and osteoporosis.

**Fig. 2:**
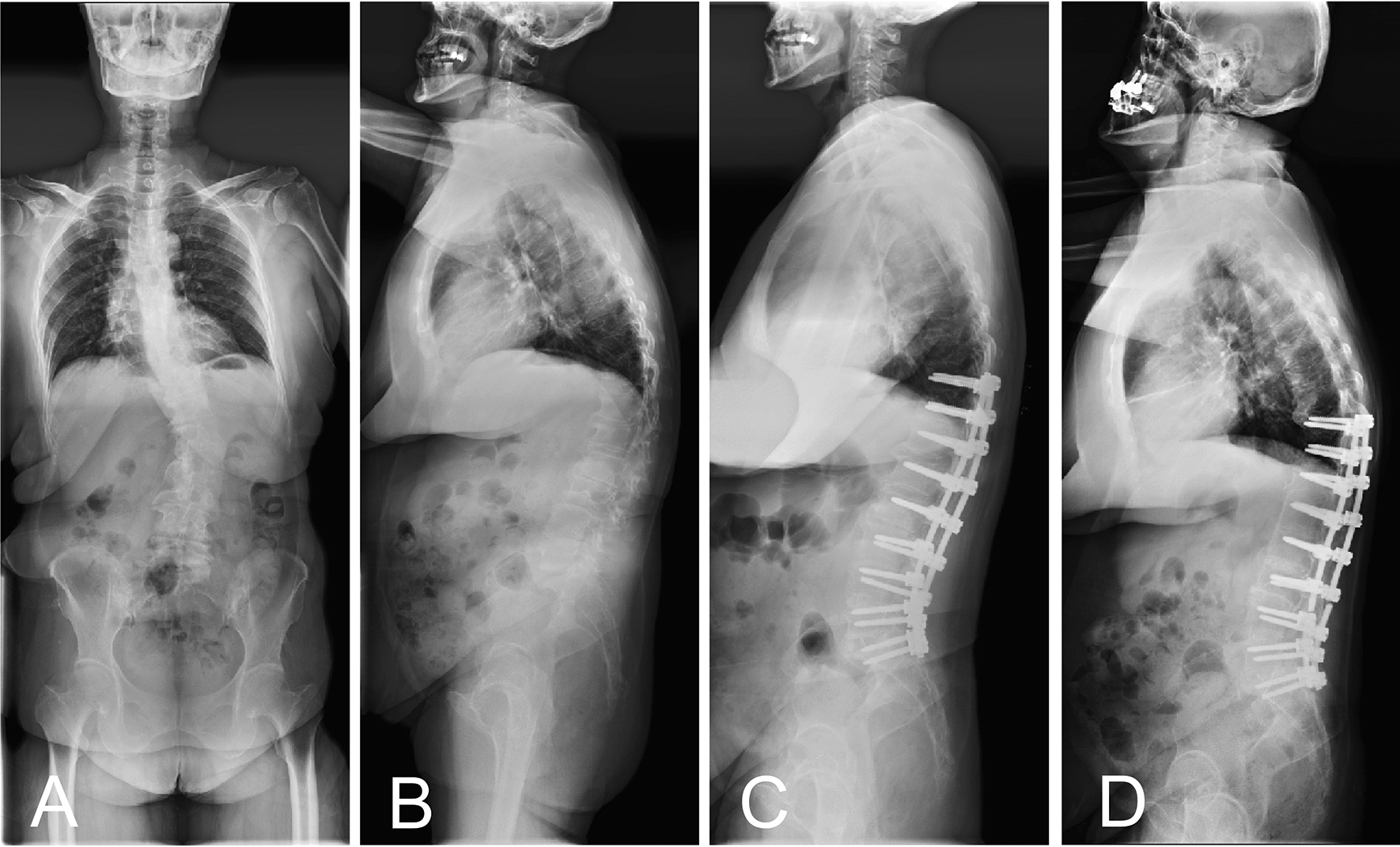
Case presentation of the impact of UIV selection on MC occurrence. Preoperative anteroposterior and lateral radiographs of a 61-year-old female with DLS and concomitant TLK (**A**, **B**). The preoperative TLK was 35.9° and the preoperative KA was located at T12. Immediate postoperative lateral radiograph showed that the fusion segment was T10-S1 **(C)**. Postoperative KA shifted to T10 following the corrective surgery and UIV was located at KA area. A 42-month postoperative lateral radiograph showed that rod fracture and PJK occurred (**D**)

**Fig. 3:**
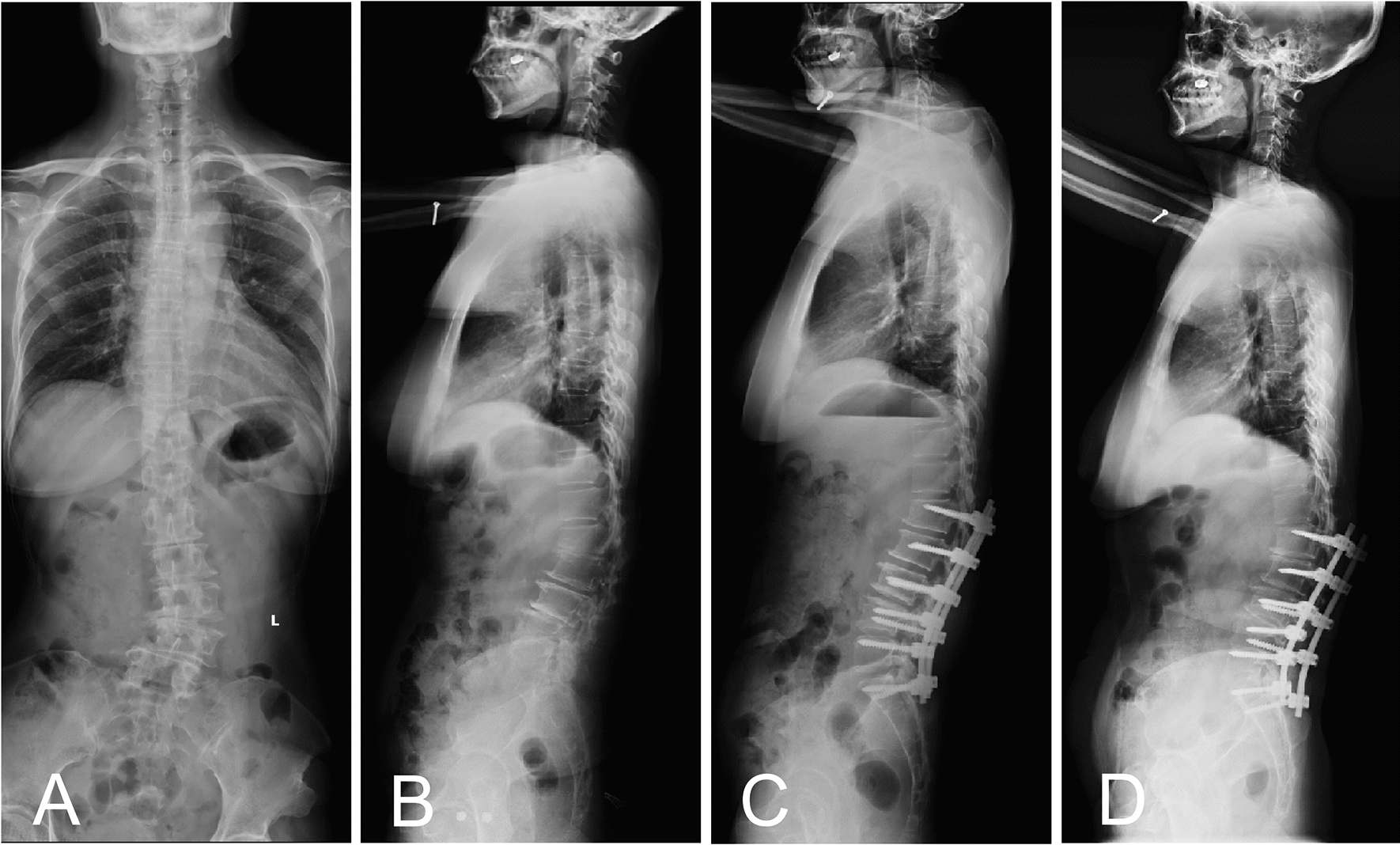
Case presentation of the impact of UIV selection on MC occurrence. Preoperative anteroposterior and lateral radiographs of a 50-year-old female with DLS and concomitant TLK (**A, B**). The preoperative TLK was 26.3° and the preoperative KA was located at L1. Immediate postoperative lateral radiograph showed that the fusion segment was L1-S1 **(C)**. TLK was retained and UIV was located at KA area. An 8-month postoperative lateral radiograph showed that PJF occurred, and revision surgery was performed (**D**)

Due to our specific patient selection, all patients exhibited a TLK > 20°, and preoperative KA were all located at the thoracolumbar junction (T10-L2). Therefore, the positional relationship with the KA inevitably needs to be considered when determining the lower thoracic UIV levels. Following the correction of TLK, the positions of KA could shift cranially, showing a tendency towards restoration to the physiological thoracic kyphosis apex. If the TLK was not adequately corrected, the KA might still stay in the thoracolumbar junction area. In this study, the presence of UIV in the KA area was closely associated with the occurrence of MC (Figs. 2, 3). This was consistent with the classical recommendations that the UIV should not be stopped at a segment with junctional kyphosis or adjacent to the KA [26, 27]. Interestingly, following the KA migration, the proportion of UIV located in the KA area increased significantly from 29.2% preoperatively to 63.1% postoperatively. It suggested that for patients with TLK, there remained a considerable risk of proximal fusion ending at the postoperative KA area. The mutual angulation between the fixed segment and the proximal unfused segment might increase the shear forces on the junctional area. Therefore, careful consideration must be given to the positional changes of the KA in the surgical planning for TLK patients when selecting the UIV. Additionally, attention must be paid to the rod contouring to prevent the formation of a new KA at the proximal fixation segment.

Accurate assessment of spinal sagittal alignment is paramount in preventing mechanical complications [28]. According to Roussouly classification, TLK could be generated from accentuating the original type 1 by increasing the lordosis on a short segment [29]. Conversely, degenerative type 1 could also emerge from other types when the kyphosing event affected the thoracolumbar area. Identifying the correspondence between PI and original “normal” spinal shape could aid in recognizing the ideal spinal morphology and preventing mechanical failure [20, 22, 30]. Previous studies by Scemama et al. [31] and Sun et al. [32] found that patients suffering from TLK with could obtain satisfactory clinical outcomes when restored type 1 or type 2. However, the patient selection in those studies were limited in a low PI population. Our study found that the proportion of Roussouly mismatch in the MC group was significant increased, which might further validate the guiding value of the Roussouly classification in the prevention of MC, particularly for patients with TLK.

In the presence of TLK, the lower lumbar curvature tended to exhibit hyperlordosis as a compensatory mechanism for the loss of LL and to maintain sagittal balance [15]. Thus, a diminished DLL might accompany a greater sagittal imbalance. In our study, patients in the MC group had a lower preoperative DLL and a higher preoperative SVA. Similarly, it has been reported in the literature that patients with preoperative sagittal imbalance are more likely to develop PJK [12, 33, 34]. For patients with TLK, the loss of DLL made the spine more prone to a global kyphosis shape, indicative of sagittal decompensation, thereby increasing the likelihood of developing MC. Generally, elderly patients with degenerative spinal deformity exhibit a more rigid spine, which reduces their ability to disperse mechanical stress. However, there was no significant difference in TLK flexibility between the MC group and the non-MC group.

The development of MC was influenced by multiple factors. Therefore, in addition to formulating surgical strategies, multidimensional preoperative assessment and management are also required for the prevention of MC. In this study, osteoporosis was also identified as an independent risk factor for MC in patients with DLS and concomitant TLK. Therefore, perioperative anti-osteoporosis treatment remained beneficial in reducing the incidence of MC.

There existed some limitations in this study. First, it was a single-center retrospective study and selection bias cannot be completely avoided. Thus, the selection of UIV is subject to the surgeon experience and preference. Second, due to our stringent patient selection, a substantial proportion of patients missing 2 year follow-up were excluded. The sample size in this study was relatively small, which may limit the statistical power and the generalizability of our findings. A multicenter study with more target patient samples is needed in the future. Third, this study primarily analyzes imaging and clinical outcomes. The thoracolumbar junction, serving as the biomechanical transition zone of the spine, is prone to degeneration and injury. The impact of TLK on MC occurrence requires further analysis through biomechanical research.

## Conclusion

In summary, UIV located in the KA area, preoperative sagittal imbalance and osteoporosis were identified as significant risk factors for MC in patients with DLS and concomitant TLK. The spinal morphological characteristics of TLK must be taken into account in preoperative evaluation and surgical planning. In particular, it is important to consider the postoperative changes in the KA position, and to avoid placing the UIV in the kyphotic region.

## Data Availability

No datasets were generated or analysed during the current study.

## Abbreviations

TLK: Thoracolumbar kyphosis
DLS: Degenerative lumbar scoliosis
ASD: Adult spinal deformity
MC: Mechanical complication
PJK: Proximal junctional kyphosis
PJF: Proximal junctional failure
DJK/F: Distal junctional kyphosis or failure
UIV: Upper instrumented vertebrae
LIV: Lowest instrumented vertebrae
KA: Kyphotic apex
LA: Lordotic apex
DLL: Distal lumbar lordosis
LL: Lumbar lordosis
SS: Sacral slope
PT: Pelvic tilt
PI: Pelvic incidence
TK: Thoracic kyphosis
TPA: T1 pelvic angle
SVA: Sagittal vertical axis
PJA: Proximal junctional angle

## References

[1] Pellise F, Bayo MC, Ruiz de Villa A, et al. The Impact of unplanned reoperation following adult spinal deformity surgery: a prospective longitudinal cohort study with 5-year follow-up. J Bone Joint Surg Am. 2024;106(8):681–9. 38630053 10.2106/JBJS.23.00242

[2] Yilgor C, Sogunmez N, Boissiere L, et al. Global alignment and proportion (GAP) score: development and validation of a new method of analyzing spinopelvic alignment to predict mechanical complications after adult spinal deformity surgery. J Bone Joint Surg Am. 2017;99(19):1661–72. 28976431 10.2106/JBJS.16.01594

[3] Yagi M, Fujita N, Tsuji O, et al. Low Bone-Mineral density is a significant risk for proximal junctional failure after surgical correction of adult spinal deformity: a propensity score-matched analysis. Spine. 2018;43(7):485–91. 28767638 10.1097/BRS.0000000000002355

[4] Oe S, Yamato Y, Hasegawa T, et al. The validation study of preoperative surgical planning for corrective target in adult spinal deformity surgery with 5-year follow-up for mechanical complications. Eur Spine J. 2022;31(12):3662–72. 36245039 10.1007/s00586-022-07420-7

[5] Teles AR, Aldebeyan S, Aoude A, et al. Mechanical complications in adult spinal deformity surgery: can spinal alignment explain everything? Spine. 2022;47(1):E1–9. 34468439 10.1097/BRS.0000000000004217

[6] Filley A, Baldwin A, Ben-Natan AR, et al. The influence of osteoporosis on mechanical complications in lumbar fusion surgery: a systematic review. N Am Spine Soc J. 2024;18:100327. 38962714 10.1016/j.xnsj.2024.100327 PMC11219986

[7] Lechtholz-Zey EA, Gettleman BS, Ayad M, et al. The effect of osteoporosis on complications and reoperation rates after surgical management of adult thoracolumbar spinal deformity: a systematic review and meta analysis. Global Spine J. 2024. 10.1177/21925682241250031. 38666610 10.1177/21925682241250031 PMC11528736

[8] Shang Z, Liu Y, Yuan H, et al. Inherited genetic predisposition and imaging concordance in degenerative lumbar scoliosis patients and their descendants. J Orthop Surg Res. 2024;19(1):494. 39169360 10.1186/s13018-024-05000-7 PMC11337562

[9] Zhang H, Du Y, Zhao Y, et al. Outcomes of Ponte osteotomy combined with posterior lumbar interbody fusion for reconstruction of coronal and sagittal balance in degenerative scoliosis. J Orthop Surg Res. 2023;18(1):904. 38017583 10.1186/s13018-023-04371-7 PMC10685520

[10] Hou X, Sun Z, Li W, et al. Upper instrumented vertebrae selection criteria for degenerative lumbar scoliosis based on the hounsfield unit asymmetry of the first coronal reverse vertebrae: an observational study. J Orthop Surg Res. 2023;18(1):819. 37907995 10.1186/s13018-023-04325-z PMC10619298

[11] Schwab FJ, Smith VA, Biserni M, et al. Adult scoliosis: a quantitative radiographic and clinical analysis. Spine. 2002;27(4):387–92. 11840105 10.1097/00007632-200202150-00012

[12] Yuan L, Zeng Y, Chen Z, et al. Degenerative lumbar scoliosis patients with proximal junctional kyphosis have lower muscularity, fatty degeneration at the lumbar area. Eur Spine J. 2021;30(5):1133–43. 33210198 10.1007/s00586-020-06394-8

[13] Zhao J, Yang M, Yang Y, et al. Proximal junctional kyphosis in adult spinal deformity: a novel predictive index. Eur Spine J. 2018;27(9):2303–11. 29508074 10.1007/s00586-018-5514-3

[14] Kawabata A, Yoshii T, Sakai K, et al. Identification of predictive factors for mechanical complications after adult spinal deformity surgery: a multi-institutional retrospective study. Spine. 2020;45(17):1185–92. 32205686 10.1097/BRS.0000000000003500

[15] Lamartina C, Berjano P. Classification of sagittal imbalance based on spinal alignment and compensatory mechanisms. Eur Spine J. 2014;23(6):1177–89. 24682355 10.1007/s00586-014-3227-9

[16] Berjano P, Lamartina C. Classification of degenerative segment disease in adults with deformity of the lumbar or thoracolumbar spine. Eur Spine J. 2014;23(9):1815–24. 24563272 10.1007/s00586-014-3219-9

[17] Moridaira H, Inami S, Takeuchi D, et al. Can we use shorter constructs while maintaining satisfactory sagittal plane alignment for adult spinal deformity? J Neurosurg Spine. 2020;34(4):589–96. 33361482 10.3171/2020.7.SPINE20917

[18] Kumar RP, Adida S, Lavadi RS, et al. A guide to selecting upper thoracic versus lower thoracic uppermost instrumented vertebra in adult spinal deformity correction. Eur Spine J. 2024;33(7):2742–50. 38522054 10.1007/s00586-024-08206-9

[19] Glattes RC, Bridwell KH, Lenke LG, et al. Proximal junctional kyphosis in adult spinal deformity following long instrumented posterior spinal fusion: incidence, outcomes, and risk factor analysis. Spine. 2005;30(14):1643–9. 16025035 10.1097/01.brs.0000169451.76359.49

[20] Sebaaly A, Gehrchen M, Silvestre C, et al. Mechanical complications in adult spinal deformity and the effect of restoring the spinal shapes according to the Roussouly classification: a multicentric study. Eur Spine J. 2020;29(4):904–13. 31875922 10.1007/s00586-019-06253-1

[21] Laouissat F, Sebaaly A, Gehrchen M, et al. Classification of normal sagittal spine alignment: refounding the Roussouly classification. Eur Spine J. 2018;27(8):2002–11. 28455623 10.1007/s00586-017-5111-x

[22] Pizones J, Moreno-Manzanaro L, Sanchez Perez-Grueso FJ, et al. Restoring the ideal Roussouly sagittal profile in adult scoliosis surgery decreases the risk of mechanical complications. Eur Spine J. 2020;29(1):54–62. 31641904 10.1007/s00586-019-06176-x

[23] Lowe T, Berven SH, Schwab FJ, et al. The SRS classification for adult spinal deformity: building on the King/Moe and Lenke classification systems. Spine. 2006;31(19 Suppl):S119-25. 16946628 10.1097/01.brs.0000232709.48446.be

[24] Schwab F, Ungar B, Blondel B, et al. Scoliosis research society-schwab adult spinal deformity classification: a validation study. Spine. 2012;37(12):1077–82. 22045006 10.1097/BRS.0b013e31823e15e2

[25] Roussouly P, Gollogly S, Berthonnaud E, et al. Classification of the normal variation in the sagittal alignment of the human lumbar spine and pelvis in the standing position. Spine. 2005;30(3):346–53. 15682018 10.1097/01.brs.0000152379.54463.65

[26] Shufflebarger H, Suk SI, Mardjetko S. Debate: determining the upper instrumented vertebra in the management of adult degenerative scoliosis: stopping at T10 versus L1. Spine. 2006;31(19 Suppl):S185-94. 16946637 10.1097/01.brs.0000232811.08673.03

[27] Silva FE, Lenke LG. Adult degenerative scoliosis: evaluation and management. Neurosurg Focus. 2010;28(3):E1. 20192655 10.3171/2010.1.FOCUS09271

[28] Bourret S, Cerpa M, Kelly MP, et al. Correlation analysis of the PI-LL mismatch according to the pelvic incidence from a database of 468 asymptomatic volunteers. Eur Spine J. 2022;31(6):1413–20. 35325301 10.1007/s00586-021-07087-6

[29] Sebaaly A, Grobost P, Mallam L, et al. Description of the sagittal alignment of the degenerative human spine. Eur Spine J. 2018;27(2):489–96. 29177554 10.1007/s00586-017-5404-0

[30] Sebaaly A, Riouallon G, Obeid I, et al. Proximal junctional kyphosis in adult scoliosis: comparison of four radiological predictor models. Eur Spine J. 2018;27(3):613–21. 28597300 10.1007/s00586-017-5172-x

[31] Scemama C, Laouissat F, Abelin-Genevois K, et al. Surgical treatment of thoraco-lumbar kyphosis (TLK) associated with low pelvic incidence. Eur Spine J. 2017;26(8):2146–52. 28180982 10.1007/s00586-017-4984-z

[32] Sun W, Wang S, Wang B, et al. Surgical treatment of Roussouly type 1 with realigning Roussouly spinal shape and improving SRS-Schwab modifier: effect on minimal clinically important difference. Eur Spine J. 2024;33(6):2486–94. 38632137 10.1007/s00586-024-08245-2

[33] Kim HJ, Bridwell KH, Lenke LG, et al. Proximal junctional kyphosis results in inferior SRS pain subscores in adult deformity patients. Spine. 2013;38(11):896–901. 23232215 10.1097/BRS.0b013e3182815b42

[34] Buell TJ, Chen CJ, Quinn JC, et al. Alignment risk factors for proximal junctional kyphosis and the effect of lower thoracic junctional tethers for adult spinal deformity. World Neurosurg. 2019;121:e96–103. 30217780 10.1016/j.wneu.2018.08.242

